# Adversarial Attack and Defense in Breast Cancer Deep Learning Systems

**DOI:** 10.3390/bioengineering10080973

**Published:** 2023-08-17

**Authors:** Yang Li, Shaoying Liu

**Affiliations:** Graduate School of Advanced Science and Engineering, Hiroshima University, Higashihiroshima 739-8511, Japan

**Keywords:** adversarial attacks, defense, breast cancer, deep learning, security

## Abstract

Deep-learning-assisted medical diagnosis has brought revolutionary innovations to medicine. Breast cancer is a great threat to women’s health, and deep-learning-assisted diagnosis of breast cancer pathology images can save manpower and improve diagnostic accuracy. However, researchers have found that deep learning systems based on natural images are vulnerable to attacks that can lead to errors in recognition and classification, raising security concerns about deep systems based on medical images. We used the adversarial attack algorithm FGSM to reveal that breast cancer deep learning systems are vulnerable to attacks and thus misclassify breast cancer pathology images. To address this problem, we built a deep learning system for breast cancer pathology image recognition with better defense performance. Accurate diagnosis of medical images is related to the health status of patients. Therefore, it is very important and meaningful to improve the security and reliability of medical deep learning systems before they are actually deployed.

## 1. Introduction

With the rapid development of information technology, artificial intelligence technology has achieved great success [1,2,3]. Deep learning is a subset of artificial intelligence that uses deep neural networks and mimics the neuronal networks in the brain via reinforcement training so that the machine can make accurate decisions autonomously [4,5,6]. The rapid development of deep learning has brought breakthroughs in many fields, such as autonomous driving, healthcare, and disease prediction [7,8,9]. In particular, the powerful recognition and processing of images based on deep learning are bringing exciting changes to radiomics [10]. Lambin [11] first introduced the concept of radiomics in 2012, referring to the extraction of high-throughput features from medical images and further employing diverse statistical analysis and data mining methods to extract and strip out key information from massive amounts of information, which is ultimately used to assist in the diagnosis, classification, or prediction of diseases [12].

Breast cancer is one of the most common malignant tumors in women, its incidence is the highest among female malignant tumors, and it is developing earlier and becoming more prevalent [13,14,15]. Clinical studies have shown that early detection and precise treatment of breast cancer can effectively reduce the risk of death in patients, thus increasing the success rate of breast cancer treatment [16,17]. Therefore, the accurate identification and diagnosis of pathological images in breast cancer clinics is crucial for patients. It can help doctors make accurate judgments and assessments of a patient’s condition to provide precise treatment for the patient’s condition. Radiomics technology provides great assistance in the adjuvant treatment and prediction of breast cancer [18,19,20]. Medical radiologists mainly use radiomics to observe the characteristics of breast pathological tissue information for the quantitative analysis of breast cancer cells, lymphocytes, and glands, to effectively diagnose breast pathology images and assess disease [21].

The rapid development of deep learning has revolutionized many fields, among which its use to assist in medical diagnosis has been groundbreaking [22,23,24]. Deep learning technology provides an effective tool for the early detection and clinical grading of breast cancer diagnoses, and there have been many studies that have applied deep learning technology to breast cancer diagnosis [25]. Spanhol et al. constructed deep learning models for the classification task of breast cancer histopathology images based on publicly available breast cancer histopathology images datasets [26]. Dhungel Net et al. proposed a fully automated deep learning system with higher accuracy for the task of detecting, segmenting, and classifying breast lumps in mammograms [27]. Benzheng Wei et al. proposed a BiCNN model for histopathological image classification methods for breast cancer. In addition, a data enhancement method that can fully preserve the image edge features of cancerous regions was proposed, and the model has good robustness and generalization with an accuracy of 97%, providing an effective aid for the clinical diagnosis of breast cancer [28]. Alom et al. proposed an inception recurrent residual convolutional neural network for the classification of breast cancer pathology images that has better classification performance compared with existing machine learning methods [29]. Anderson et al. evaluated two different deep learning methods to classify benign and malignant datasets based on breast lesions and compared them with lesion-based radiomics computer-aided diagnosis methods [30]. Vandenberghe et al. constructed a new deep learning approach to identify the HER2 biomarker for breast cancer to identify high-risk misdiagnosis cases and thus assist in clinical decision making for breast cancer diagnosis [31]. Khan S.U. et al. proposed a deep learning framework for highly accurately classifying and identifying breast cancer cell images based on the transfer learning method [32]. Han et al. proposed a new deep learning model for breast cancer multi-classification that has remarkable performance in breast cancer multi-classification tasks with an average accuracy of 93.2%, providing an effective tool for breast cancer multi-classification clinical diagnosis [33]. Wang et al. constructed a classifier based on a semi-automatic segmentation method to classify and identify microcalcification and breast masses in the breast, and the accuracy of the classification results was greater than 85%, which may be of great clinical significance for the detection and treatment of breast cancer [34]. Saha M. et al. proposed a new deep learning supervised model for detecting WSI mitotic images of breast cancer pathology with 92% precision that can help physicians to perform better evaluations and grading diagnoses of breast cancer [35].

Deep learning has greatly improved the accuracy of breast cancer pathology image recognition and classification, and it has also provided effective diagnostic aids in the early detection and graded treatment of breast cancer [36]. However, the security of medical systems based on medically assisted diagnosis is more important than in other deep learning systems. Adversarial attacks are the biggest potential security vulnerability in medical imaging deep learning systems. This can lead to the misdiagnosis of a patient’s condition and thus miss the time for treatment. Therefore, the security and reliability of medical deep learning systems is a topic of concern. From the beginning of the design of medical deep learning systems, safety and reliability should be prioritized. The application of deep learning to medicine is to better assist physicians in improving the efficiency and accuracy of medical diagnosis, and if the security of deep systems is compromised, it is extremely harmful to both physicians and patients. Improving the safety and reliability of healthcare systems is a very important topic [37,38,39]. Therefore, in this paper, we studied the security of a deep learning system based on breast cancer pathological images. Specifically, we used an adversarial attack algorithm to generate adversarial images for attacking breast cancer deep learning systems as well as to construct a defense model against such attacks. The contributions of this paper are as follows:

1. We used transfer learning to build a deep learning system that can accurately identify benign and malignant breast tumor pathology images, and the model achieved an average recognition accuracy of 98.72%.
2. We used an adversarial attack algorithm to attack the trained model so that the deep learning system misclassified the breast cancer images, which reduced the model’s recognition accuracy for breast cancer images from 98.90% to 10.99%. It was demonstrated that the above breast cancer deep learning system has security vulnerabilities and can be affected by adversarial attack.
3. To address the security vulnerabilities in the deep learning system for breast cancer pathology images, we built a defense deep learning system for breast cancer pathology images with better defense performance. The defense model could defend against the adversarial attack algorithm, and the recognition accuracy for breast cancer images decreases from 96.70% to 27.47% in the face of the same adversarial attack algorithm.

## 2. Preliminaries

In this section, we introduce adversarial attacks and defenses against adversarial attacks in deep learning systems. 

### 2.1. Adversarial Attack

Deep neural networks have shown powerful capabilities for image recognition and classification [40,41,42]. However, Szegedy found that deep neural networks have fatal weaknesses in image classification tasks when adding perturbations to the input image that are difficult to detect with the eye, which can cause the models to generate classification errors [43]. An adversarial sample is defined as follows.

Suppose *x* is the input data, f is the deep learning model, and the classification result of the model is *f*(*x*). If there is a perturbation *ϵ* satisfying the following equation, we can refer to *x* as the adversarial sample of the model *f*.

*f* (*x* + *ϵ*)*!* = *f* (*x*)

Since Szegedy discovered this phenomenon, it has attracted many researchers to study adversarial attacks and to pay more attention to the security and reliability of deep learning systems. Goodfellow et al. proposed the fast gradient sign method (FGSM) to generate adversarial samples by performing only one gradient step, and this method reduces the computational cost of generating adversarial samples [44]. Momentum I-FGSM builds on FGSM, wherein the gradient update direction is stabilized, and the convergence process is optimized to improve the transference of the adversarial samples [45]. The basic iterative method (BIM) expands on the FGSM by performing multiple small-step iterations and trimming the pixel values of the result after each step to ensure that the result is in the ϵ neighborhood of the original image [46]. Projected gradient descent (PGD) can be treated as a multi-step iteration of the FGSM, taking one small step at a time, and each iteration clips the perturbation to the specified range [47]. DeepFool defines sample robustness and model robustness and can accurately compute deep classifier perturbations in large-scale datasets to reliably quantify the robustness of classifiers [48].

According to different attack algorithms, adversarial attacks can be gradient-based, optimization-based, or adversarial-network-based [49]. A gradient-based adversarial attack is obtained by calculating the gradient from the input image during the model training process and subsequently updating the input image by calculating the loss function and obtaining the adversarial image. There are many gradient-based adversarial attack methods, among which are the algorithms FGSM, I-FGSM, and PGD. An optimization-based adversarial attack refers to continuously calculating and reducing the loss function between the predicted and true values of the sample data during the training process of the model and, subsequently, by adjusting and updating the parameters in the model during the backward transfer process, and finally obtaining the adversarial image. Optimization-based adversarial attacks include JMSA, C&W, and L-BFGS [50,51,52]. An adversarial-network-based adversarial attack is based on an adversarial network (GAN) as the skeleton, and the adversarial image is obtained by optimizing the loss function between the predicted data and the real data [53]. The adversarial samples generated based on the GAN have higher realism and high similarity, and the adversarial attack methods are AdvGAN, AdvGAN++, and Natural GAN [54,55,56].

According to different attack environments, adversarial attacks can be white-box attacks, black-box attacks, or gray-box attacks [57]. White-box attacks know the network structure and parameters of the model and other information. Black-box attacks do not know the internal structure and parameters of the model and can only attack the model through the output. Gray-box attacks are aware of a part of the model’s information but do not fully grasp all the information of the model.

According to the purpose of the adversarial attack, it can be divided into targeted and untargeted attacks [58]. The purpose of targeted attacks is to make the model not only misclassify the sample but also specify the type into which the input data will be misclassified. Contrary to a targeted attack, an untargeted attack aims to simply cause the model to misclassify the input data.

### 2.2. Defense against Adversarial Attack

Faced with the threat of adversarial samples, researchers have proposed some methods of adversarial sample defense to protect deep learning models. Most white-box attacks obtain an adversarial sample by computing the gradient of the model, so if the gradient of the model cannot be computed, the attack will be ineffective. Gradient masking changes the model to some extent, thus making the gradient useless and resisting the adversarial sample well. Florian Tramèr et al. proposed powerful single-step attack strategies and integrated adversarial training by migrating perturbation inputs from other pre-trained models, thus decoupling the two processes of adversarial sample generation and model parameter training and increasing the diversity of perturbations in the training process [59]. Dongyu Meng proposed a framework for MagNet, including independent detector networks and a reformer network, where the detector network is used to detect normal original and adversarial samples, thus making it difficult for the adversarial samples to attack the neural network model [60].

## 3. Methodology

**Figure 1:**
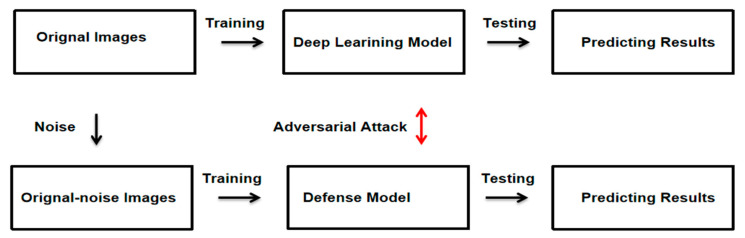
Adversarial attack and defense in breast cancer deep learning system.

The method proposed in this paper consists of three parts. Firstly, benign pathological images of breast cancer and malignant pathological images are used as input images, and the two types of images are trained based on the transfer learning method with Desnet-121 as the skeleton to obtain a deep learning model that can accurately identify the two types of images. The trained model is attacked with adversarial attacks to generate adversarial images, which makes the model misclassify the adversarial images, thus also leading to a decrease in the accuracy of the model for image recognition. To overcome this problem, we propose a defensive approach against adversarial attacks, building up a more secure, reliable, and robust defense deep learning system. The methods are shown in Figure 1.

### 3.1. Deep Learning System Construction Based on Transfer Learning

**Figure 2:**
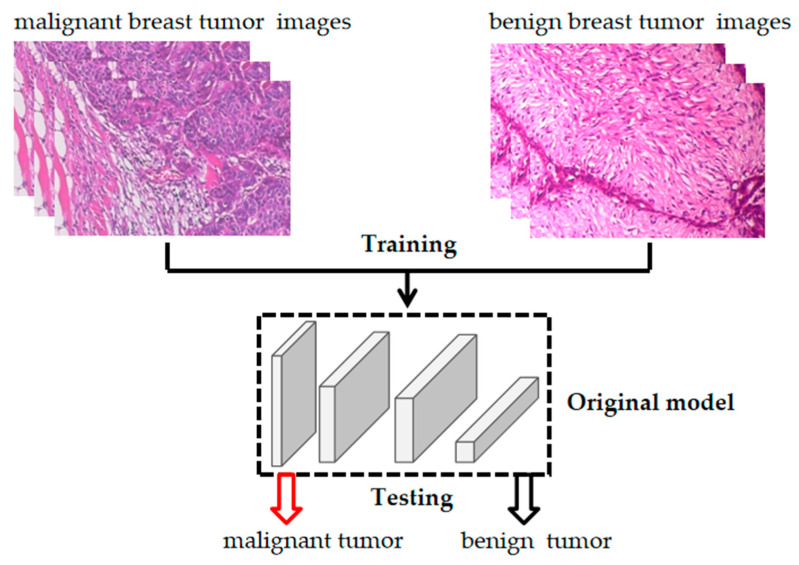
Construction of a deep learning model for breast cancer.

We constructed a deep learning system for breast cancer using the transfer learning approach. The model construction consists of two parts: model training and performance testing (Figure 2). In this section, we will discuss these questions in detail.

#### 3.1.1. Datasets

The breast cancer pathology image data in this paper were obtained from the breast cancer pathological database (BreakHis) [61]. This dataset is anonymous and publicly available for non-commercial studies on breast cancer images. This dataset contains 644 benign breast tumor pathology images and 903 malignant breast tumor pathology (breast cancer) images. We divided the whole dataset into three parts: a training set, validation set, and test set for the breast cancer deep learning model construction and adversarial attack and defense experiments (Table 1). The training set was used to train the deep learning model, the validation set was used to tune the hyperparameters of the model, and the test set was used to test and evaluate the performance of the trained deep learning model.

#### 3.1.2. Transfer Learning from the DenseNet121 Model

Transfer learning is a common approach in deep learning, whereby trained models are used to accomplish new tasks by exploiting the similarity between models and targets. By using transfer learning, we can take an existing trained model, migrate it to our task, and then fine-tune the model for our task-specific requirements to save training costs and time and quickly achieve the task requirements. Due to the small amount of data from the breast cancer pathology images, we adopted a transfer learning approach using the DenseNet121 model pre-trained on chest X-rays to achieve better results [62]. The DenseNet network was designed to connect each layer directly to its preceding layers to achieve the reuse of features and to effectively solve the gradient disappearance problem while designing each layer of the network to be particularly narrow, requiring only a very small number of feature maps to be learned, thus substantially reducing the number of parameters [63]. We performed a data augmentation operation on the dataset, and the size of the input image was cropped to 224 × 224 × 3. The deep learning model was trained with 400 epochs using the Adam optimizer with a small batch size of 32 and an initial learning rate of 0.001 [64].

### 3.2. Adversarial Attack on Breast Cancer Deep Learning System

The fast gradient sign method (FGSM) is a gradient-based method for generating adversarial samples that maximize the loss function in the opposite direction of the decreasing gradient during the data propagation and updating of a neural network. The expression of FGSM is shown below, where *x* is the input sample, *y* is the label corresponding to sample *x*, *x_adv_* is the adversarial sample, *θ* is the weight parameter of the model, the manually set perturbation parameter of the model is *ϵ*, and the loss function of the model is *J*().

*x_adv_* = *x* + *ϵ*· *sign* (*∇x J*(*θ, x, y*))

One of the security risks of medical image deep learning systems is that the original breast cancer images are modified into benign tumor images by maliciously tampering with medical images, thus making the models misclassify them. This leads to the misdiagnosis of the patient’s condition, thus making the patient miss the best time for treatment. In order to test the security of the breast cancer deep learning system, we conducted an adversarial attack on the trained model using breast cancer images as the research object and added subtle interference to the test set, which caused the deep learning model to misclassify the images. We use the FGSM algorithm to attack the trained model and generate adversarial samples that are difficult to distinguish with the eye.

### 3.3. Defense against Adversarial Attack in Breast Cancer Deep Learning System

**Figure 3:**
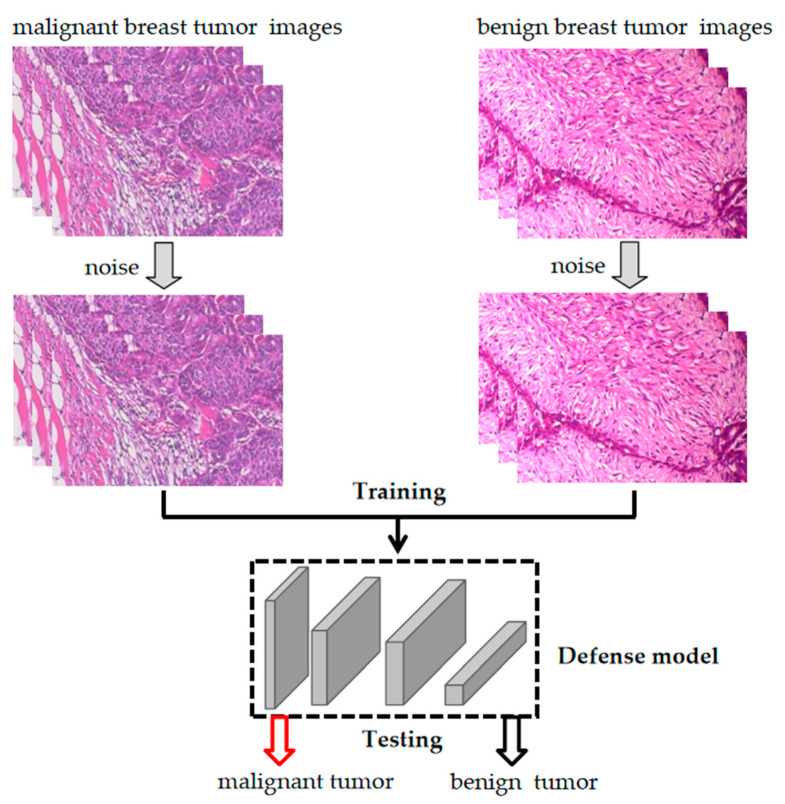
Construction of a defense deep learning model for breast cancer.

We used an adversarial attack to attack the deep learning model by slightly altering the original image’s pixel to generate an adversarial image, thus fooling the model and making it misclassify the image. If we added noise to the original image before training the model, the new image was trained so that the model could obtain more feature information from the noisy image (Figure 3).

**Figure 4:**
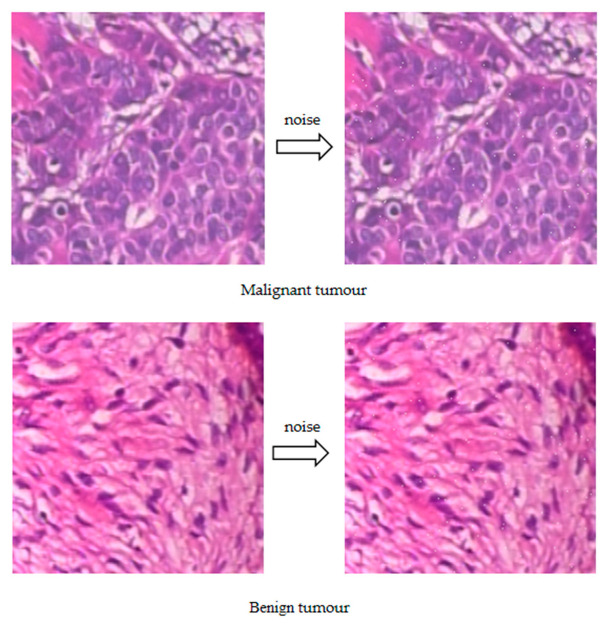
Characteristic results of Gaussian noise image.

We used the noisy images as input data to train and build a defense deep learning system with the same model and parameters as the original model and test the performance of the defense model with the original test set without added noise. Gaussian noise is noise whose probability density function obeys a Gaussian distribution. In the construction of the adversarial defense model, we chose to add Gaussian noise to the original image, and the comparison between the Gaussian noise image and the original image was as follows (Figure 4). We normalized the original image so that the pixel values were distributed between 0 and 1, then created a matrix of noisy images with a Gaussian distribution, and finally added the noise to the original image to obtain a new image with noise. To better display the Gaussian noise image, we partially zoomed in on the image.

### 3.4. Metrics for Evaluating the Performance of Breast Cancer Deep Learning Systems

In this study, the performance of the breast cancer deep learning system was evaluated using the metric of accuracy. The accuracy metric was used to measure the overall correctness of the model’s classifications. True positives (TPs) represented the number of breast cancer images that were correctly identified as breast cancer images. False positives (FPs) indicated the number of benign tumor images that were incorrectly classified as breast cancer images. True negatives (TNs) represented the number of benign tumor images that were accurately identified as benign tumor images. Finally, false negatives (FNs) indicated the number of breast cancer images that were mistakenly classified as benign tumor images.

$$Accuracy=\frac{TPs+TNs}{TPs+TNs+FPs+FNs}$$

### 3.5. Instrument

The experiments were coded in Python 3.8 with Pytorch on a personal computer with an NVIDIA GeForce 3070 graphics processing unit (GPU) with 8 Gb of random access memory (RAM) (NVIDIA Corp, Santa Clara, CA, USA).

## 4. Results

This section shows the experimental results.

### 4.1. The Accuracy of Breast Cancer Deep Learning Systems

The performance test results of the deep learning model are shown in Table 2. The accuracy of the original deep learning model was 98.72%, and the accuracy of the defense model was 98.08% on the same test set, which indicates that adding noise to the input image does not affect the recognition and classification ability of the defense model for the image, and both the defense model and the original model can accurately identify the medical images of benign and malignant breast tumors with good recognition and classification abilities.

**Figure 5:**
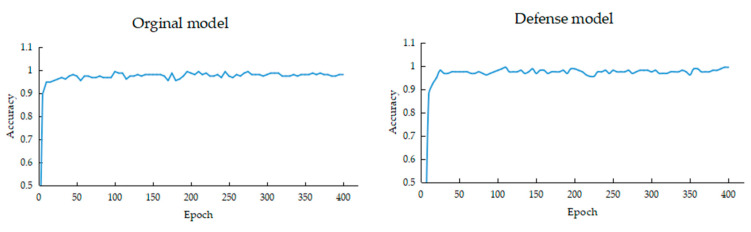
Accuracy of deep learning models for breast cancer.

Figure 5 shows the change in the accuracy of the models as the number of training, increases. Because the dataset had few images, we trained the deep learning model using the transfer learning method, and the accuracy of the model increased rapidly, and the performance reached saturation quickly.

### 4.2. The Recognition Accuracy of Breast Cancer Deep Learning Systems after Adversarial Attack

To reveal the threat of adversarial attacks on breast cancer deep learning systems and better simulate the security risk of real-world deep systems based on breast cancer images, we used breast cancer images as the research object and attacked the pre-trained model with the FGSM adversarial attack algorithm and then tested the defense capabilities of the original model and the defense model against the adversarial attacks, and the results are shown in Table 3. When the original model was attacked by the adversarial attack algorithm, the recognition accuracy of the model decreased from 98.90% to 10.99%, which indicates that the model was successfully attacked by the adversarial attack algorithm and that the attack can severely damage the performance of the model. Similarly, when the defense model was attacked by the adversarial attack algorithm, the recognition accuracy of the model decreased from 96.70% to 27.47%, which indicates that the defense model was also successfully attacked by the adversarial attack algorithm, but the recognition accuracy of the defense model increased by 16.48% compared with the original model when facing the same adversarial attack, thus indicating that the defensive model has a certain defensive capability against the adversarial attack algorithm compared with the original model. The defense model demonstrated better security, reliability, and robustness performance.

**Figure 6:**
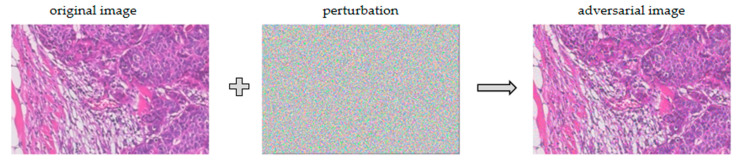
Characteristic results of adversarial image and original image.

To better illustrate the adversarial attack, we compared the original image with the adversarial image, as shown in Figure 6. We took a breast cancer image as an example and used the FGSM algorithm to perform the adversarial attack on the deep learning model. The adversarial image was generated with a slight perturbation of the original image, and it was difficult for us to distinguish the difference between the two images with our eyes, but the deep learning model misclassified them, which further illustrates that the adversarial attack was a great threat to the deep learning system.

## 5. Discussion

We tested and studied a deep learning system for pathological images of breast cancer. In contrast with previous studies on breast cancer deep learning, our research focused on the security and reliability of deep learning systems based on breast cancer pathology images, and we demonstrated through adversarial attacks that deep learning systems based on breast cancer pathology images have security vulnerabilities and can be easily attacked.

We trained a deep learning model that can accurately identify benign and malignant breast tumors using transfer learning with an average accuracy of 98.72%, but the model is susceptible to attacks by adversarial attack algorithms. To better reveal the security risks that exist in real-world breast cancer deep learning systems, we demonstrated that the model’s recognition accuracy of images dropped from 98.90% to 10.99% after being attacked using breast tumor images as the study object.

To better defend against this adversarial attack, we superimposed Gaussian noise onto the input images at the beginning of the training phase of the model and then retrained the model and tested the performance of the defense model with the same test set as the original model, and the model achieved a recognition accuracy of 98.08% for the original test set, which indicates that superimposing noise on the input data does not affect the recognition accuracy of the whole model. However, when we attacked the defense model with the same adversarial attack algorithm, we found that the accuracy of the model only decreased from 96.70% to 27.47%, which is a 16.48% improvement compared with the original model, indicating that the model has a defensive performance against the adversarial attack algorithm and can effectively defend against an adversarial attack. While this may not appear as a substantial advancement, it indisputably demonstrates the defensive model’s superior security and robustness in the face of adversarial attack challenges.

## 6. Conclusions

In this work, we studied adversarial attack and defense in breast cancer deep learning systems. In contrast with previous studies, we demonstrated the excellence of using deep learning in medical-image-assisted diagnosis while revealing its limitations. More importantly, breast cancer deep learning systems are vulnerable to adversarial attacks that misclassify medical images. To address the threat of adversarial attack on the security of breast cancer deep learning systems, we propose a method that can defend against adversarial attack, thus effectively reducing the success rate of adversarial attack and improving the security and reliability of the deep learning system.

Undoubtedly, there remains a need for further research and development to effectively apply the breast cancer deep learning model in real world scenarios. Adapting the model to real world environments will require addressing various challenges and potential threats. Continual refinement and exploration of novel defensive mechanisms will be necessary to fortify the deep learning model’s defenses against increasingly sophisticated adversarial attacks. In future work, we will continue to study the security and reliability of the medical deep learning system. We also hope that more researchers will pay attention to the security of medical deep learning systems.

## Figures and Tables

**Table 1:** Division of breast pathology images in the dataset.

Dataset	Training Set	Validation Set	Test Set	Total
Benign	515	64	65	644
Malignant	722	90	91	903

**Table 2:** The accuracy of two deep learning models on the same test set.

Metric	Original Model	Defense Model
Accuracy (%)	98.72	98.08

**Table 3:** The accuracy of two deep learning models subjected to adversarial attack.

Attack	Accuracy (%)	
	Original Model	Defense Model
**No attack**	98.90	96.70
**FGSM attack**	10.99	27.47

## Data Availability

The data presented in this study are available upon request from the corresponding author.
